# Read-Level Error Characterization of Rolling-Circle Amplification-Based Nanopore Sequencing of the Circular DNA Virome

**DOI:** 10.3390/v18070704

**Published:** 2026-06-26

**Authors:** Florencia Martino, Kakhangchung Panmei, Dylan Duchen, David L. Thomas, Abraham J. Kandathil, Steven J. Clipman

**Affiliations:** 1 Division of Infectious Diseases, Johns Hopkins University School of Medicine, Baltimore, MD 21287, USA; fmarti34@jh.edu (F.M.); kpanmei1@jh.edu (K.P.); dthomas@jhmi.edu (D.L.T.); abrahamjk@jhmi.edu (A.J.K.); 2 Department of Pathology, Yale School of Medicine, New Haven, CT 06519, USA; dylan.duchen@yale.edu

**Keywords:** circular DNA viruses, *Anelloviridae*, M13mp18 phage, long-read sequencing, viral metagenomics, rolling-circle amplification, plasma virome

## Abstract

Oxford Nanopore technology enables cost-effective, portable, long-read analyses of pathogen genomes. Accurate detection and interpretation of small circular viral genomes, including *Anelloviridae*, remain challenging due to limited base-level error quantification in rolling-circle amplification (RCA)-derived datasets. Here, we characterized read-level sequencing error profiles using M13mp18, a 7.2 kb circular phage genome, subjected to 1X and 3X shearing during library preparation. M13mp18 DNA was serially diluted into pooled anellovirus-positive plasma DNA extracts. Using custom error-analysis pipelines, we quantified mismatch, insertion, and deletion rates and evaluated consensus reconstruction accuracy across simulated sequencing depths. Since metagenomic viromes contain mixtures of related genomes and uneven coverage across taxa, depth-normalized subsampling was used to assess the precision of read-level error estimates under heterogeneous coverage. Across four benchmarked datasets, per-base error rates ranged from 0.018 to 0.022 errors per aligned base. Complete M13mp18 reference reconstruction was achieved at input levels ≥ 4.6 log_10_ copies, and consensus sequences reached 100% identity at depths ≥ 15X when sufficient reads were available. Below 4.6 log_10_ input copies, recovery was inconsistent. These findings provide a controlled empirical characterization of read-level error behavior in RCA-derived nanopore sequencing and support the interpretation of circular DNA virome data generated in complex metagenomic backgrounds.

## 1. Introduction

Oxford Nanopore Technologies (ONT) has become a prominent platform for viral genomics, providing portable, relatively low-cost sequencing with real-time data generation and long reads capable of capturing within-host diversity in complex metagenomic samples [1]. Clinical metagenomics increasingly requires pathogen-agnostic sequencing approaches capable of detecting both known and unexpected viruses, even in low-biomass samples [1]. The recent deployment of R10.4.1 flow cells, incorporating a dual-reader-head design and duplex basecalling, has reduced indel rates and improved consensus accuracy, particularly in homopolymer-rich regions [2,3]. For example, in Influenza A virus sequencing, R10.4.1 demonstrated superior performance over R9.4.1, resolving problematic motifs in 90% of cases compared to 60%, thus enabling more reliable viral genome reconstruction [4].

The family *Anelloviridae* encompasses a diverse array of single-stranded circular DNA (ssDNA) viruses that typically range from 1.6 to 3.9 kilobases in size [5]. These viruses are found ubiquitously in various biological systems, including human blood plasma [6,7]. Although anelloviruses have not been definitively linked to specific diseases, increasing evidence suggests that anellovirus abundance may reflect host immune status [8,9,10]. Higher anellovirus loads have been associated with immunosuppression, transplantation outcomes, and chronic viral infections, supporting their potential use as biomarkers of immune function [8,9,10]. Consequently, accurate detection and characterization of anelloviruses may have value not only for virome studies but also for future investigations examining associations between anellovirus dynamics and host immunity.

Due to their circular genomes, sequencing protocols often employ RCA to enrich viral DNA. RCA has been widely applied for sequencing small circular DNA molecules, including viruses in humans and plants, as well as plasmids, where its ability to generate concatemeric amplicons substantially improves sensitivity and genome recovery [11,12,13]. Although effective for increasing sensitivity, RCA generates concatemeric products and uneven coverage, complicating genome assembly, taxonomic classification, and variant detection [11]. While RCA applications have largely focused on Illumina sequencing, RCA’s compatibility with long-read platforms provides an additional advantage by producing reads that traverse entire concatemers and preserve the physical continuity of sequence variants. We previously developed and validated an RCA–nanopore sequencing protocol optimized for anelloviruses isolated from plasma samples, with a focus on maximizing read yield, specificity, and multiplexing [14]. Building on that framework, the present study characterizes read-level error profiles generated during nanopore sequencing of RCA-amplified circular DNA using updated flow cell chemistry and basecalling models.

While improvements in ONT chemistry and basecalling have enhanced consensus-level accuracy broadly, systematic quantification of raw per-base errors introduced by RCA in rich metagenomic backgrounds remains unexplored. The plasma virome, defined as the totality of viral populations circulating in the blood, represents a dynamic ecosystem shaped by complex host–virus interactions and characterized by extensive genomic diversity [7,15]. Accurate interpretation of metagenomic sequencing data in this context remains challenging, as viral communities frequently exhibit high sequence heterogeneity, uneven genomic coverage, and ambiguous read mapping among closely related variants [15]. Under these conditions, differences observed at the read level may reflect true biological variation, cross-mapping between homologous regions, or sequencing error, and many circulating variants lack complete or well-curated references. Characterizing read-level error profiles under defined amplification and sequencing conditions is therefore important for interpreting metagenomic virome datasets. While previous studies have described performance characteristics of RCA and nanopore sequencing independently [2,3,11,13,16], the cumulative error introduced across the complete RCA–nanopore workflow remains poorly defined. This characterization is especially relevant for circular templates amplified by RCA, where concatemeric products, uneven depth, and homologous genomic regions can complicate the interpretation of read-level variation. To characterize these effects, we evaluated the composition of read-level basecalling errors introduced during nanopore sequencing of RCA-amplified circular DNA, using a clonal spike-in with a defined reference sequence. As a controlled surrogate for anellovirus genomes, we used the commercially available circular single-stranded M13mp18 DNA phage genome, which was selected due to its similar genome size and architecture to that of anelloviruses (single-stranded DNA). M13mp18 DNA was serially diluted into DNA extracts from human plasma, followed by RCA enrichment to model realistic and biologically complex sample backgrounds.

## 2. Methods

### 2.1. Sample Preparation

M13mp18 circular single-stranded DNA (NEB, Ipswich, MA, USA; Cat. No. N4040S) was serially diluted (1:10) in DNA extracted from the pooled plasma of seven anellovirus-positive participants, previously characterized by PCR and metagenomic sequencing [17]. Plasma DNA was extracted from 200 µL of input plasma and eluted in 60 µL of nuclease-free water to maintain a consistent complex background across all dilutions.

Copy numbers for M13mp18 were estimated based on DNA mass and genome size (7.2 kb), where 1 ng corresponds to approximately 2.7 × 10^8^ molecules. DNA concentrations (ng/µL) were converted to copies/µL accordingly. For each dilution, total input copies were calculated based on a 30 µL reaction volume corresponding to the starting material used for downstream exonuclease treatment, expressed as copies per reaction on a log_10_ scale.

These diluted M13mp18 inputs, ranging from 5.6 to 1.6 log_10_ copies per reaction, were treated to remove linear or nicked dsDNA, after which RCA was performed on each dilution. The resulting concatemer products were fragmented using the Bioruptor under the following settings: 15 s on/30 s off for 3 cycles (3X) to generate distinct fragment length distributions, as previously described [14]. A set of pure M13mp18 (cssDNA) (11.6 log_10_ copies per reaction) underwent the same RCA and post-amplification shearing workflow at 1X and 3X cycles.

Libraries were then prepared from the fragmented products for sequencing on the Oxford Nanopore Technologies (ONT) platform [14]. Input concentrations ranged from 5.6 to 1.6 log_10_ copies per RCA reaction, providing a broad input range for downstream sequencing analyses.

### 2.2. Nanopore Sequencing & Quality Control

**Figure 1:**
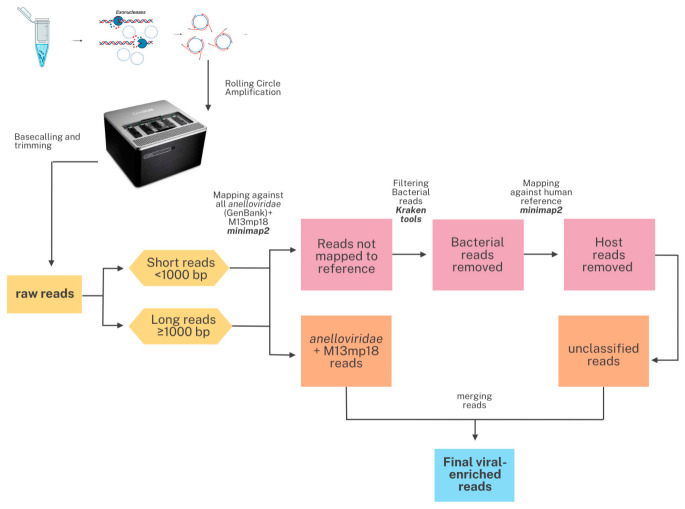
Workflow for read processing and decontamination. Raw reads were split into short (<1000 bp) and long (≥1000 bp) categories, followed by mapping against the family *Anelloviridae* and M13mp18 reference. Reads that mapped to either reference were retained, while unmapped reads were sequentially filtered to remove bacterial contaminants using Kraken2 and host-derived sequences (human genome) using Minimap2. After decontamination, filtered reads were merged with those initially mapped to the family *Anelloviridae* and M13mp18, yielding the final clean read set used for downstream analyses.

Library preparations were performed using the ONT ligation kit (SQK-LSK114) and the PCR barcode kit (EXP-PBC096) (Oxford Nanopore Technologies, Oxford, UK). Sequencing was conducted on the ONT GridION platform with the R10.4.1 flow cell chemistry. Basecalling was performed using Dorado v1.0.0 (https://github.com/nanoporetech/dorado, accessed on 25 March 2026) (SUP model) in simplex mode and specifying the EXP-PBC096 barcode kit. Adapter and barcode trimming were applied during demultiplexing using the “barcode both-ends” flag. Quality filtering was implemented post-demultiplexing using SeqKit (Q ≥ 10) to separate high- and low-quality reads. Adapter trimming was primarily performed via Dorado; Porechop v0.2.4 (https://github.com/rrwick/Porechop, accessed on 25 March 2026) was used to validate trimming consistency. Trimmed reads were then mapped against a custom reference database containing genomes from members of the family *Anelloviridae* and M13mp18 using Minimap2 v2.24 [18]. Reads ≥ 1000 bp were mapped with the map-ont preset, and reads < 1000 bp were mapped with the sr preset. Secondary alignments were suppressed using --secondary=no. Reads that mapped to either reference were retained. Unmapped reads were filtered in successive steps to remove bacterial contaminants using Kraken2 v2.0.7-beta [19], followed by Krakentools (extract_kraken_reads.py) with taxid 2 and --include-children [20], and host-derived reads were removed by mapping against the human reference genome GRCh38 [GenBank: GCF_000001405.40], using Minimap2 with the same read-length-specific presets. These filtered unmapped reads were subsequently merged with those that originally mapped to the family *Anelloviridae* and M13mp18 to generate a final clean set of reads for downstream analysis (Figure 1). The taxonomic composition of the complex plasma background was profiled at the species level with Kraken2 [19] and Bracken v2.9 [21]. Unless otherwise indicated, software tools were run using recommended default parameters.

### 2.3. Read Mapping and Depth Simulation

All FASTQ files were split into “long” (≥1 kb) and “short” (<1 kb) read fractions using SeqKit v2.9.0 [22] and mapped to the M13mp18 reference genome (GenBank: X02513.1) using Minimap2 v2.28 [18]. The `map-ont` preset was used for long reads and the `sr` preset for short reads, with secondary alignments suppressed (`--secondary=no`).

To avoid spurious alignments, the M13mp18 reference was truncated to nucleotide positions 1–4550, excluding the *lacZ* fragment cloning site derived from *E. coli*. Any read spanning beyond coordinate 4550 was discarded to prevent spurious mapping unrelated to the viral M13mp18 genome. Sequencing depth normalization was simulated at a logarithmic scale using BBnorm, starting at 10X and extending up to the maximum sequencing depth obtained for each sample [23].

### 2.4. Error Analysis

Base-level sequencing errors, including mismatches, insertions, and deletions (indels), were quantified using a custom Python v3.11.15 tool (https://github.com/MERIDAIN-Lab/ONT-RCA-error-profiler/, accessed on 25 March 2026). This tool compares each aligned read by parsing the Compact Idiosyncratic Gapped Alignment Report (CIGAR) string, which encodes how well reads align to a reference. For example: ‘M’ signifies an aligned base match (which may be a sequence match or mismatch), ‘I’ an insertion relative to the reference, ‘D’ a deletion, ‘=’ an exact match, ‘X’ a mismatch, and ‘S’ a soft-clipped region that is not present in the reference. Final BAMs with simulated depths were also analyzed with SAMtools v1.21 [24] and Qualimap v2.2.2 [25] for comparison purposes. We implemented a custom script in addition to these tools because they summarize alignment statistics but do not directly quantify mismatches and indels at the per-base level. Coverage and depth metrics were calculated separately using SAMtools v1.21 [24]. Here, we define the per-base error rate as the proportion of aligned positions that are mismatches or indels, computed from the SAM ‘NM’ and CIGAR (and/or MD) tags. Specifically, error_rate = NM/(M + I + D), where NM is the total number of edit operations (mismatched bases + inserted bases + deleted bases), and M, I, and D are the CIGAR counts for aligned columns (*M*, or equivalently the sum of = and X in extended CIGAR format), insertions, and deletions, respectively. Soft-clipped bases are excluded from the denominator. We restricted our analysis to primary alignments (no secondary) and did not apply a minimum base-quality (Phred) filter. For clarity, per-base error rates are expressed as fractional values (errors per aligned base), where 0.01 corresponds to a 1% error rate.

### 2.5. Consensus Accuracy

To assess the accuracy of consensus sequences generated at each simulated depth level, we applied the iVar tool v1.4.4 [26] to construct one M13mp18 consensus per sample using a majority-rule approach with a 60% threshold, requiring a minimum coverage of 10 reads and base quality ≥ Q20. Positions not meeting these criteria were called N. The resulting consensus FASTA sequences were then aligned to the M13mp18 reference (GenBank: X02513.1) using BLASTn v2.17.0 [27]. Percent identity and query coverage were extracted to quantify reconstruction accuracy across simulated depths.

## 3. Results

### 3.1. Sequencing Output and M13mp18 Recovery Across Input Conditions

We quantified sequencing yield and M13mp18 recovery accuracy under three experimental conditions: (i) purified M13mp18 DNA under the 1X sheared condition, (ii) purified M13mp18 DNA under the 3X sheared condition, and (iii) a five-point serial dilution (5.6 log_10_ to 1.6 log_10_ input copies) of M13mp18 into an anellovirus-positive human plasma DNA extract background, all processed under the 3X sheared condition. The 1X sheared condition library yielded 99,420 reads (median length 1011 bp), whereas the 3X sheared condition produced 1,112,512 reads (median length 753 bp). In the dilution series, read counts ranged from 578,918 to 2,978,052, with median read length decreasing from 1499 bp (lowest input) to 899 bp (highest input). Sequencing performance metrics for each run are summarized in Supplementary Table S1, and read-length versus basecalling-quality distributions are shown in Supplementary Figure S1. The taxonomic composition of the plasma DNA background is summarized in Supplementary Figure S2.

### 3.2. Mapping-Based Reference Recovery Across Input Conditions

Mapping-based M13mp18 recovery across the dilution series was complete at input levels ≥ 4.6 log_10_ input copies per reaction, when evaluated as 100% breadth at ≥10X depth across the retained 4550-bp reference interval. Below 4.6 log_10_ input copies per reaction, M13mp18 recovery was inconsistent. Some lower-input samples yielded partial M13mp18 coverage under relaxed criteria, whereas at least one intermediate dilution did not yield a recoverable signal, indicating stochastic recovery near the detection boundary.

### 3.3. Read-Level Error Composition Across Sample Types and Simulated Depths

Read-level sequencing errors were quantified using the ONT-RCA error profiler for all experimental conditions, which compares primary aligned reads against the M13mp18 reference to identify mismatches and indels. Across the four benchmarked datasets with sufficient M13mp18-mapped reads, total per-base error rates ranged from 0.018 to 0.022 errors per aligned base (Table 1). In the plasma DNA matrix, total error rates were 0.019 for both the 4.6 and 5.6 log_10_ input copies per reaction. For pure M13mp18 controls, the total error rate was 0.018 and 0.022 per base aligned under the 1X and 3X sheared conditions, respectively. Error composition was broadly similar across datasets, with mismatches representing the most frequent error type (0.008–0.011 per aligned base), followed by deletions (0.006–0.007) and insertions (0.004–0.005) [Table 1]. Depth-targeted subsampling was used as a sensitivity analysis to evaluate the stability of read-level error estimates under reduced sampling using the ONT-RCA error profiler (Supplementary Figure S3). As expected, lower-depth subsamples showed greater uncertainty, whereas higher-depth subsamples produced more stable mean estimates. For cross-condition benchmarking of read-level error composition, analyses focused on positions with ≥30X mapped depth, where error-rate estimates were more stable across subsampled datasets.

This coverage cutoff was applied only for read-level benchmarking and was not used as a requirement for consensus reconstruction. Error-rate estimates obtained from depth-simulated subsampling were similar across analytical approaches, including the ONT-RCA error profiler, Qualimap v2.3, and SAMtools v1.21 (Supplementary Figure S4).

**Figure 2:**
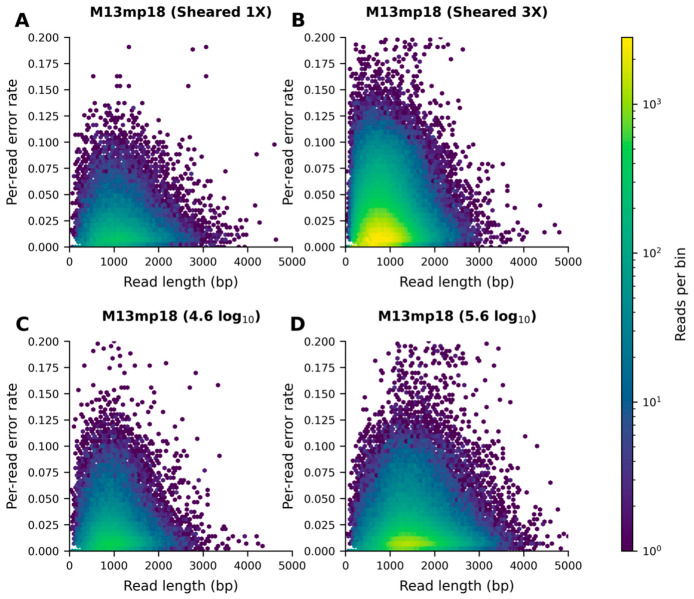
Per-read error rate as a function of read length across experimental conditions. Error rate was computed for each read from its alignment to the M13mp18 reference as the fraction of aligned bases affected by substitutions or insertion/deletion events; soft-clipped segments were excluded. Across all datasets, the highest read densities were observed among reads approximately 500–1500 bp in length, with most reads exhibiting relatively low per-read error rates. Hexagonal binning summarizes read density, with the color scale indicating the log-transformed read count per hexagon. Panels show (**A**) pure M13mp18 sheared 1X, (**B**) pure M13mp18 sheared 3X, (**C**) M13mp18 spiked into an anellovirus-positive plasma DNA matrix at 4.6 log_10_ input copies, and (**D**) the corresponding plasma matrix dilution at 5.6 log_10_ input copies.

Read-level error rates were also evaluated as a function of read length. Per-read error rates (mismatches + insertions + deletions; excluding soft-clipped bases) were computed from primary alignments and visualized as read density using hexagonal binning (Figure 2). Across datasets, most reads clustered between approximately 500–1500 bp and showed low per-read error rates, whereas fewer reads were observed at greater lengths (Figure 2). Across datasets, approximately 45% of indels occurred in homopolymer regions, in line with known nanopore error patterns.

### 3.4. Consensus Sequence Accuracy and Completeness as a Function of Sequencing Depth

Consensus sequences achieved complete reconstruction of the analyzed 4550 bp reference interval with 100% identity to the M13mp18 reference at depths ≥ 15X across datasets with sufficient reads, i.e., with 0 mismatches and 0 gaps in the evaluated alignments in this defined control system (Supplementary Figure S5). At 10X depth, consensus sequences were near-complete but showed occasional deviations depending on the dataset, with percent identity ranging from 99.8% to 100% (0–5 mismatches, 0 gaps) and aligned reads ranging from 2542 to 4550 bp.

## 4. Discussion

Accurate interpretation of metagenomic virome data requires understanding the read-level error profile generated under defined experimental conditions. Metagenomic surveillance and virome studies often involve complex biological matrices, motivating empirical error benchmarking under realistic sample backgrounds [3]. Here, we characterized the read-level error profiles generated by RCA combined with ONT sequencing in a defined circular DNA spike-in model within a complex virome background. Across the benchmarked conditions, total per-base error rates ranged from 0.018 to 0.022 errors per aligned base (1.8–2.2%), consistent with prior long-read reports and low despite concatemeric RCA products [2,16,28]. In this controlled M13mp18 system, consensus sequences reached 100% identity at depths ≥ 15X when sufficient reads were available, indicating that read-level errors were effectively resolved during consensus generation under the evaluated conditions.

Virome samples frequently contain mixtures of closely related viruses, uneven coverage across genomes, and homologous genomic regions that can complicate read mapping and variant interpretation [15]. Under these conditions, differences observed at the read level may reflect sequencing artifacts, cross-mapping between related genomes, or genuine biological variation. In addition, consensus reconstruction may not always be feasible when reference sequences are incomplete or when closely related variants coexist within the same sample. Consequently, quantitative characterization of read-level error profiles provides an interpretive baseline for evaluating read-level variation in RCA-derived metagenomic datasets. Increasing sequencing depth did not reduce the intrinsic read-level error rate, but it did improve the precision of error-rate estimates, which is particularly relevant for benchmarking and comparative analyses across datasets. The ≥30X depth threshold was therefore used only as an internal analytical filter for comparing read-level error composition across subsampled datasets and should not be interpreted as a sequencing requirement for consensus recovery.

While nanopore sequencing has traditionally exhibited higher error rates than short-read platforms, advances such as the R10.4.1 flow cells and Dorado basecalling have markedly improved accuracy [2,16,29,30]. In our data, the per-base mismatch and indel rates ranged from 0.018 to 0.022 errors per aligned base, reflecting the high performance achieved with the latest chemistry and basecalling algorithms [2,31].

A similar RCA–-nanopore approach has recently been shown to achieve error profiles compatible with the accurate detection of single-nucleotide variants and copy number changes in circulating tumor DNA [30].

Per-read error profiles were visualized across the observed read-length range. Most reads clustered between approximately 500 and 1500 bp and showed low error rates, whereas longer reads were less abundant in the benchmarked datasets, consistent with cumulative error over longer templates and exposure to structurally complex regions [32]. Read-length distributions differed between shearing conditions, with longer reads observed in the 1X than in the 3X sheared condition control. Lower input may also contribute through longer RCA concatemers. Base-level error rates in the plasma-matrix samples mirrored those of pure M13mp18 controls, suggesting that the complex virome background did not alter read-level error profiles under the evaluated conditions.

These results support the use of this controlled spike-in model to evaluate RCA–-nanopore read-level error behavior and consensus recovery under defined experimental conditions. Several considerations should be noted when interpreting these findings. A limitation of this study is the lack of independent experimental replicates for each condition (i.e., separate RCA reactions and library preparations). Therefore, the reported error rates and consensus recovery observations reflect within-run performance and do not quantify between-run, -library or -RCA variability. In addition, we did not perform a formal quantification of chimeric molecules or other structural library artifacts. In RCA-derived circular templates sequenced with long reads, split and supplementary alignments may arise from expected origin-spanning or concatemer-derived mappings as well as from true artifactual fusions, preventing unambiguous classification in the present experimental design. A rigorous analysis of structural artifacts would require a dedicated experimental and computational framework and was beyond the scope of this read-level error characterization study.

Since M13mp18 is a simple, clonal genome with limited diversity compared to anelloviruses, read mapping is inherently easier. In natural virome datasets, higher genetic diversity and the presence of closely related variants may further complicate consensus reconstruction. Accordingly, the consensus-depth observations reported here should not be interpreted as universal requirements for circular DNA viruses or metagenomic backgrounds, particularly because this benchmark used a clonal control and a retained 4550-bp analysis region.

At very low input levels, recovery was inconsistent and should be interpreted cautiously, as the apparent signal at extreme dilutions may reflect stochastic recovery and, in some cases, cross-mapping rather than a true signal. Cross-mapping was detected within the *lacZ* segment; this region was excluded from downstream analyses, yielding a retained analysis region of 4550 bp (closer to anelloviruses, ~2.8–3.9 kb). Accordingly, the empirical detection limit represents a conservative upper bound. The plasma pool used consisted of seven samples from people who inject drugs, a population with exceptionally diverse anellovirus communities [14]. The background analyzed here, therefore, represents one of the most challenging scenarios, and the thresholds reported should be regarded as an upper bound rather than an optimistic detection limit.

By modeling a complex circular DNA virome background with a defined M13mp18 spike-in, this study provides a controlled empirical description of read-level error composition in RCA-derived nanopore data. The observed consensus recovery at sufficient depth provides an internal validation of the dataset but should not be interpreted as a universal reconstruction threshold. Overall, these results provide a technical reference for interpreting read-level variation in RCA-enriched circular DNA sequencing studies, including future longitudinal studies of individual anellovirus communities (“anellomes”) and their relation to host immune status, and define priorities for future work that includes independent RCA reactions, library preparations, sequencing runs, and additional circular viral targets.

## Figures and Tables

**Table 1:** Sequencing yield and dataset-level per-base error composition across M13mp18 input conditions and fragmentation protocols.

M13mp18 Sample Condition	Raw Reads	Clean Reads	Mapped Reads	SNV Rate	Insertion Rate	Deletion Rate	Total Per-Base Error Rate
Pure M13mp18 input, sheared 1X	99,420	99,419	48,362	0.009	0.004	0.006	0.018
Pure M13mp18 input, sheared 3X	1,112,512	1,112,495	491,130	0.011	0.005	0.007	0.022
5.6 log_10_ M13mp18 input copies in plasma DNA matrix, sheared 3X	578,918	321,524	205,856	0.009	0.004	0.006	0.019
4.6 log_10_ M13mp18 input copies in plasma DNA matrix, sheared 3X	1,620,640	468,905	67,362	0.008	0.004	0.007	0.019

Rates were computed as (error counts)/(total aligned bases), where aligned bases include matched/mismatched positions and indel events. Rates are reported separately for SNVs (mismatches), insertions, and deletions, together with the total error rate (SNV + insertion + deletion). Soft-clipped bases were excluded from both the numerator and denominator. Values are rounded to three decimal places.

## Data Availability

The scripts supporting this study are available on GitHub https://github.com/MERIDAIN-Lab/ONT-RCA-error-profiler/ (accessed on 25 March 2026). The Anellome analysis pipeline used in this study is available at https://github.com/Clipman-Lab/Anellome_pipeline (accessed on 25 March 2026). Raw nanopore reads and processed BAM files were deposited in NCBI SRA under BioProject accession PRJNA1355149 with SRA experiment accessions SRX31303792–SRX31303798.

## Supplementary Materials

The following supporting information can be downloaded at Zenodo https://zenodo.org/records/17387676 (accessed on 25 March 2026), Figure S1: Read length versus read quality distributions for each sequencing run; Figure S2: Species-level taxonomic profiles obtained with Bracken for the M13mp18 dilution series in plasma DNA matrix; Figure S3: Precision of read-level base error estimates under depth-targeted subsampling of M13mp18 datasets; Figure S4: Read-level error-rate estimates across depth-targeted subsampling of M13mp18 datasets using three analytical approaches; Figure S5: Consensus reconstruction accuracy and completeness as a function of sequencing depth; Table S1: Sequencing performance metrics across Nanopore sequencing conditions; Table S2: Sequencing performance metrics for M13mp18-mapped reads and mapping-depth summary across benchmarked conditions.
